# Association of serum omentin levels with cardiac autonomic neuropathy in patients with type 2 diabetes mellitus: a hospital-based study

**DOI:** 10.1186/s12933-015-0303-3

**Published:** 2015-10-14

**Authors:** Chan-Hee Jung, Sang-Hee Jung, Bo-Yeon Kim, Chul-Hee Kim, Sung-Koo Kang, Ji-Oh Mok

**Affiliations:** Division of Endocrinology and Metabolism, Department of Internal Medicine, Soonchunhyang University College of Medicine, #170 Jomaru-ro, Wonmi-gu, Bucheon-si, Gyeonggi-do 420-767 Republic of Korea; Department of Obstetrics and Gynecology, Cha University School of Medicine, Bundang, Korea

**Keywords:** Omentin, Cardiac autonomic neuropathy, Vascular complication, Type 2 diabetes mellitus

## Abstract

**Background:**

Whereas a few studies have reported associations of serum omentin levels with subclinical atherosclerosis in patients with diabetes, little information is available with respect to the associations of serum omentin levels and diabetic microvascular complications. The aim of this study was to investigate the relationships of serum omentin levels and vascular complications including cardiac autonomic neuropathy (CAN) in patients with type 2 diabetes mellitus (T2DM).

**Methods:**

We recruited 97 patients who evaluated complications of diabetes. CAN was assessed by five standard cardiovascular reflex tests according to Ewing’s protocol. Diabetic nephropathy (DN), retinopathy (DR), and peripheral neuropathy (DPN) were evaluated. Serum omentin levels were assessed by ELISA. Atherosclerotic burden was evaluated by measuring the brachial-ankle pulse wave velocity (baPWV) and ankle brachial index (ABI).

**Results:**

The prevalence of CAN increased borderline significantly across the omentin tertiles (p = 0.05) and CAN point increased significantly and progressively across the omentin tertiles (p = 0.013). The prevalence of other microvascular complications (DPN, DN, and DR) did not differ among omentin tertiles. The mean levels of baPWV also increased significantly and progressively across the omentin tertiles (p = 0.002). Serum omentin levels were significantly positively correlated with CAN point (p = 0.004) and borderline significantly correlated with baPWV (p = 0.05) after multivariate adjustment. Regarding linear regression analysis for CAN point, univariate regression analysis demonstrated that CAN point associated with omentin, diastolic blood pressure (DBP) and hsCRP. Multiple regression analysis revealed that omentin levels, together DBP and baPWV correlated with CAN point. This present study suggests that serum omentin levels may be independently associate with CAN in patients with T2DM.

## Background

It has been hypothesized that diabetic microvascular and macrovascular complications have common pathogeneses. Insulin resistance or adipocytokines may be common underlying causes of diabetes vascular complications [1]. Moreover, adipose tissue dysfunction and an altered fat distribution might be involved in insulin resistance and atherosclerosis [2].

Omentin is a protein composed of 313 aminoacids, which is produced exclusively in the stromal cells of visceral adipose tissue [3, 4]. The known effects of omentin include insulin-sensitizing and, anti-inflammatory activities beneficial to vascular health. In several clinical studies, serum levels of omentin were reported to be reduced during dysmetabolic states [5–7]. Serum omentin levels also are negatively correlated with BMI, metabolic risk factors and inflammatory markers, and positively correlated with serum adiponectin and high density lipoprotein-cholesterol (HDL-C) levels [8–11]. However, contradictory results also have been reported regarding the association of serum omentin with the above-mentioned metabolic risk factors [12, 13].

One of the most overlooked of all of the complications of diabetes is cardiac autonomic neuropathy (CAN), which is a significant cause of morbidity and mortality in patients with diabetes [14, 15]. Although the mechanisms by which CAN leads to increased mortality remain unclear, poor glycemic control, duration of diabetes and other cardiovascular disease (CVD) risk factors such as hypertension and smoking play key roles in the development and severity of CAN. However, strict control of these risk factors does not abolish the risk of CAN, suggesting that other factors that contribute to CAN are yet to be identified [16, 17].

Whereas a few studies have reported associations of serum omentin levels with carotid atherosclerosis or arterial stiffness as an index of subclinical atherosclerosis in patients with diabetes, very few studies have investigated the associations of serum omentin levels with microvascular complications. Moreover, to our knowledge, no study has investigated the relationship between serum omentin and CAN in patients with T2DM.

Therefore, in the present study, we examined the relationship among serum omentin levels, subclinical atherosclerosis markers, and microvascular complications including CAN in patients with T2DM.

## Methods

### Patients

We recruited 146 patients with diabetes who were evaluated for microvascular complications at the diabetes clinic of Soonchunhyang University Bucheon Hospital from April 2012 to February 2014. Among them, those with type 1 diabetes, and those with severe illness, such as heart failure, liver cirrhosis with ascites, severe infection, malignancy, uncontrolled hyperthyroidism or a history of arrhythmia, and were not available fasting serum samples or clinical data from medical records were excluded. Finally, this study included 97 patients with T2DM (men 56, women 41, mean age: 57.6 years). We reviewed detailed demographic data, biochemical data, and clinical and treatment histories from patient medical records. The smoking status of the subjects was classified as being a non-smokers or smokers (former or current). All patients were informed of the purpose of the study and written consent was obtained. The study was approved by the Institutional Review Board of Soonchunhyang University School of Medicine, Bucheon Hospital.

### Measurement of serum omentin

Blood samples were taken after overnight fasting. Serum was separated and stored at −80 °C until analyzed. Serum omentin levels were measured using a commercially available enzyme-linked immunosorbent assay (ELISA; R&D Systems, Minneapolis, MN, USA). Serum insulin and C-peptide levels were measured by radioimmunoassay (Immunotech, Prague, Czech Republic).

### Cardiac autonomic neuropathy and microvascular complications

CAN was assessed by an autonomic function test (AFT). CAN was assessed by five standard cardiovascular reflex tests according to Ewing’s protocol [18]. The patients were asked to fast for 12 h before the test, and to avoid taking antihypertensive agent such as beta-blocker, caffeine, nicotine, or antihistamines. Three of these measurements mainly assess parasympathetic function : heart rate responses to deep breathing (beat-to-beat variation), to standing (30:15 ratio), and to the Valsalva maneuver. The other two tests mainly assess sympathetic function: blood pressure responses to standing and to a sustained handgrip. The heart rate response to deep breathing, standing, and the Valsalva maneuver were assessed automatically from ECG recordings using the DICAN evaluation system (Medicore Co. Ltd, Korea). The results of each of the above five tests for the detection of CAN were classified into one of three categories based on the severity of abnormality detected, and each of them was given a definite point value as described by Bellavere et al. [19]. The severity of CAN was quantified from the summation of the points obtained from each of the five tests, where each test was given a point value of 0, 0.5, or 1 if it yielded normal, borderline, or abnormal values, respectively. Consequently, the minimum and maximum numbers of autonomic neuropathy points were 0 and 5, respectively. CAN was defined as the presence of at least two abnormal tests or an autonomic neuropathy points total ≥2.

Diabetic nephropathy (DN) was defined using albuminuria, which was measured by radioimmunoassay (Immunotech). An albumin excretion rate (AER) <20 μg/min or urine albumin <30 mg/g creatinine were the criteria for normoalbuminuria; an AER in the range of 20–200 μg/min or urine albumin 30–300 mg/g creatinine, microalbuminuria; and an AER ≥200 μg/min or urine albumin ≥300 mg/g creatinine as overt proteinuria. Patients were considered to have nephropathy if they had microalbuminuria or overt proteinuria.

Diabetic retinopathy (DR) was evaluated by experienced ophthalmologists while the patients’ pupils were dilated. If needed, fluorescein angiography was performed. Patients were classified as normal, or as having non-proliferative or proliferative retinopathy; patients were considered to have retinopathy if they were in the non-proliferative or proliferative stage.

Diabetic peripheral neuropathy (DPN) was diagnosed in subjects displaying two or more of the following features: typical subjective neuropathic symptoms determined using the Michigan Neuropathy Screening Instrument (MNSI), insensitivity to a 10-g monofilament, abnormal pin-prick sensation, and abnormal current perception threshold (CPT), or a nerve conduction study. CPT was performed with a Neurometer CPT/C (Neurotron, Inc., Baltimore, MD, USA).

An automated device (VP-1000; Colin, Japan) was used to measure arterial PWV and the ABI. Insulin resistance status was evaluated by the homeostasis model assessment-insulin resistance (HOMA-IR) index. The HOMA-IR was calculated using the formulae: [fasting insulin (uIU/mL) × fasting blood glucose (mmol/L)]/22.5. The HOMA-IR score was available in only 89 patients not receiving exogenous insulin.

### Statistical analysis

Statistical analysis was performed using SPSS 14.0 (SPSS Inc, Chicago, IL, USA). Data are reported as mean ± standard deviation (SD) for normally distributed variables, as median (interquartile range) for non-normally distributed variables or as number of participants (percentages). Statistical differences in demographic and clinical characteristics between groups by gender were evaluated by means of Chi square test or Fisher’s exact test for categorical variables and Student’s *t* test or Wilcoxon’s ranksum test for continuous variables. Before the t test, Shapiro–Wilk test for normality and Levene’s homogeneity of variance test was conducted. Non-normally distributed variables, that is, omentin, hsCRP, triglyceride and HOMA-IR, were natural-logarithm-transformed before analysis. The significance of the mean differences in parameters among the tertiles of omentin levels was evaluated with one-way ANOVA. Post-hoc comparison for tertiles of omentin groups was performed with the adjusted p-value using Tukey’s HSD method.

The correlation of serum omentin levels and other clinical variables was assessed by Pearson’s correlation coefficient or Spearman’s rank correlation as appropriate. In addition, partial correlations were computed after adjustment for age, mean PWV, and CAN points. To determine the particular aspects of anthropometric, biochemical, and metabolic parameters that are related to CAN point, simple linear regression was conducted for each explanatory variable. On the result of simple linear regression, multiple linear regression analyses were performed to check the original relationship of each variable and CAN point when the other variables were adjusted. The significance of the relationship between CAN point and each explanatory variable was evaluated with a t-test for each regression coefficient. An F test was used to test the significance of the proportion of variance in CAN point (R^2^) that was explained in the model, the regression model which included the subset of explanatory variables that were at least marginally significant (p < 0.15).

## Results

### Clinical characteristics of the participants

The 97 patients consisted of 56 men and 41 women; their anthropometric parameters, clinical characteristics, and omentin levels are presented in Table 1. The mean age of the total participants was 57.6 years, and the mean duration of diabetes was 9.1 years. The mean body mass index (BMI) was 24.8 kg/m^2^. The mean level of serum omentin was 530 ± 302 ng/mL and did not differ between men and women. Only one man was treated with beta-blocker and the prevalence of prescribed medication of beta-blocker was not different between men and women. Of the 97 patients, 38 (42.2 %) were defined as having DPN and 30 (36.6 %) as having CAN. The prevalence of DN was 12 (13.3 %) and the prevalence of DR was 27 (30 %). The prevalence of DPN was higher in women than in men (56.8 vs. 32.1 %, p = 0.034). Regarding the lipid profile, higher total cholesterol and HDL-C and lower triglyceride were found in women than in men.

**Table 1:** General characteristics of the participants

	Total (n = 97)	Men (n = 56)	Women (n = 41)	p value
Anthropometric parameters				
Age	57.6 ± 8.2	56.7 ± 7.6	58.9 ± 8.9	0.181
Duration of DM (years)	9.1 ± 6.0	9.4 ± 6.5	8.9 ± 5.4	0.875
Body mass index (kg/m^2^)	24.8 ± 2.7	25.2 ± 2.2	24.3 ± 3.2	0.125
WC (cm)	86.5 ± 8.6	87.9 ± 8.0	84.5 ± 9.2	0.185
Hypertension, n (%)	39 (40.2 %)	23 (41.8 %)	16 (39 %)	0.948
Systolic BP (mmHg)	122.8 ± 14.1	120.8 ± 10.0	125.5 ± 18.1	0.419
Diastolic BP (mmHg)	74.9 ± 8.6	76.2 ± 8.6	73.2 ± 8.4	0.093
Current smoking, n (%)	19 (20.2 %)	19 (34.6 %)	0	<0.001
Alcohol intake, n (%)	30 (31.9 %)	27 (49.1 %)	3 (7.7 %)	<0.001
Biochemical and metabolic parameters				
Omentin (ng/mL)	530 ± 302	527 ± 354	534 ± 214	0.375
HbA1_C_ (mmol/mol) (%)	55 (7.2)	53 (7)	57 (7.4)	0.128
FPG (mmol/L)	7.4 ± 2.0	7.3 ± 1.8	7.5 ± 2.3	0.921
Total cholesterol (mmol/L)	4.0 ± 0.7	3.9 ± 0.7	4.2 ± 0.7	0.015
LDL-cholesterol (mmol/L)	2.3 ± 0.6	2.2 ± 0.6	2.5 ± 0.6	0.061
HDL-cholesterol (mmol/L)	1.3 ± 0.3	1.2 ± 0.3	1.3 ± 0.3	0.021
Triglycerides (mmol/L)	1.3 (0.8–1.9)	1.5 (1.0–2.0)	1.0 (0.7–1.8)	0.042
hsCRP (mg/dL)	0.07 (0.05–0.12)	0.08 (0.05–0.12)	0.07 (0.05–0.13)	0.698
Creatinine (μmol/L)	97 ± 115	97 ± 18	97 ± 168	0.861
Urinary albumin (mg/g)	16.1 ± 27.4	16.3 ± 29.0	15.8 ± 25.6	0.351
Insulin (fasting) (uIU/mL)	9.2 ± 4.9	8.9 ± 4.5	9.7 ± 5.4	0.628
C-peptide (fasting) (ng/mL)	2.2 ± 1.1	2.3 ± 0.9	2.2 ± 1.3	0.668
HOMA-IR	2.97 ± 1.62	2.80 ± 1.41	3.23 ± 1.87	0.506
ABI	1.14 ± 0.11	1.12 ± 0.14	1.15 ± 0.05	0.280
PWV (cm/s)	1493 ± 240	1480 ± 211	1511 ± 276	0.697
DPN, n (%)	38 (42.2 %)	17 (32.1 %)	21 (56.8 %)	0.034
CAN, n (%)	30 (36.6 %)	20 (40.1 %)	10 (30.3 %)	0.462
Diabetic nephropathy, n (%)	12 (13.3 %)	7 (13.5 %)	5 (13.2 %)	0.925
Diabetic retinopathy, n (%)	27 (30.0 %)	14 (26.4 %)	13 (35.1 %)	0.513
Treatment modality				0.135
No medication, n (%)	4 (4.1)	4 (7.1 %)	0	
OHA, n (%)	85 (87.6)	50 (89.3 %)	35 (85.4 %)	
OHA + insulin, n (%)	5 (5.2)	2 (3.6 %)	3 (7.3 %)	
Insulin only, n (%)	3 (3.1)	0	3 (7.3 %)	
ACEI/ARB	32 (33 %)	20 (35.7 %)	12 (29 %)	0.654
Beta-blocker	1 (1.03 %)	1 (1.8 %)	0	0.691
Statin	60 (62 %)	33 (58.9 %)	27 (65.9 %)	0.629

Data are shown as mean ± SD, median (interquartile range) or as n (%)

*DM* diabetes mellitus, *WC* waist circumference, *BP* blood pressure, *HbA1*_*C*_ hemoglobin A1_C_, *FPG* fasting plasma glucose, *LDL* low density lipoprotein, *HDL* high density lipoprotein, *hsCRP* high-sensitivity C-reactive protein, *HOMA-IR* homeostasis model assessment-insulin resistance, *ABI* ankle-brachial index, *PWV* pulse wave velocity, *OHA* oral hypoglycemic agent, *DPN* diabetic peripheral neuropathy, *CAN* cardiac autonomic neuropathy, *DN* diabetic nephropathy, *DR* diabetic retinopathy, *OHA* oral hypoglycemic agent, *ACEI* angiotensin converting enzyme inhibitor, *ARB* angiotensin receptor blocker

### Comparisons of clinical variables according to the tertiles of omentin levels

The participants were divided into three groups according to serum omentin levels. The clinical parameters according to the omentin tertile are shown in Table 2. The baPWV representing arterial stiffness significantly increased as the levels of omentin increased across the tertiles (1381 vs. 1490 vs. 1605 cm/s, p = 0.002). The mean values of anthropometric indices and metabolic variables such as blood glucose and lipid profile except total cholesterol did not differ significantly among omentin tertiles. There were no significant differences in the prevalence of ACEI, ARB or statin intake among the serum omentin tertiles. The prevalence of CAN showed an increasing trend across omentin tertiles (20.7 vs. 39.3 vs. 52 %, p = 0.05, p for trend = 0.017) and CAN points increased significantly and progressively across the omentin tertiles (p = 0.013) (Table 2).

**Table 2:** The comparisons of clinical variables according to the tertile of omentin levels

	1st tertile	2nd tertile	3rd tertile	P value (P for trend)
Omentin (ng/mL)	353 ± 36	459 ± 35	770 ± 418	<0.001
Age	54.8 ± 6.0	55.8 ± 8.3	62.1 ± 8.3	<0.001
Men/women	20/12	19/13	17/16	0.652
Duration of DM (years)	9.1 ± 5.7	9.9 ± 6.8	8.4 ± 5.5	0.779
Body mass index (kg/m^2^)	24.8 ± 2.7	25.6 ± 2.5	24.1 ± 2.8	0.071
WC (cm)	83.6 ± 8.6	88.1 ± 9.1	87.4 ± 8.0	0.160
Hypertension (%)	20/12(37.5)	19/13(40.6)	18/14(43.8)	0.878
Systolic BP (mmHg)	120.0 ± 13.9	121.9 ± 9.6	126.2 ± 17.6	0.209
Diastolic BP (mmHg)	75.3 ± 9.7	73.3 ± 7.1	76.1 ± 8.8	0.415
Current smoking, n (%)	7 (21.9)	7 (22.6)	5 (16.1)	0.785
Alcohol intake, n (%)	12 (37.5)	9 (29)	9 (29)	0.706
HbA1_C_ (mmol/mol) (%)	53 (7)	56 (7.3)	54 (7.1)	0.652
FPG (mmol/L)	6.9 ± 1.1	8.0 ± 2.3	7.2 ± 2.2	0.070
Total cholesterol (mmol/L)	3.8 ± 0.5	4.2 ± 1.1	3.4 ± 1.0	0.051
LDL-cholesterol (mmol/L)	2.2 ± 0.4	2.5 ± 0.7	2.3 ± 0.7	0.120
HDL-cholesterol (mmol/L)	1.2 ± 0.3	1.3 ± 0.3	1.3 ± 0.3	0.144
Triglycerides (mmol/L)	1.3 (0.8–1.3)	1.3 (0.8–2.0)	1.2 (1.0–1.8)	0.786
hsCRP	0.07 (0.04–0.12)	0.09 (0.06–0.13)	0.07 (0.06–0.13)	0.359
Creatinine (μmol/L)	80 ± 9	124 ± 194	88 ± 27	0.648
Urinary albumin (mg/g)	9.2 ± 9.0	17.7 ± 27.9	21.8 ± 37.4	0.561
Insulin (fasting) (uIU/mL)	9.5 ± 4.8	9.9 ± 5.9	8.3 ± 3.7	0.558
c-peptide (fasting) (ng/mL)	2.3 ± 1.2	2.3 ± 1.2	2.1 ± 0.9	0.705
HOMA-IR	2.89 ± 1.45	3.45 ± 2.04	2.61 ± 1.26	0.293
ABI	1.13 ± 0.17	1.14 ± 0.05	1.14 ± 0.07	0.222
PWV (cm/s)	1381 ± 194	1490 ± 202	1605 ± 265	0.002 (p < 0.001)
CAN points	1.1 ± 0.8	1.3 ± 0.8	1.9 ± 1.3	0.013 (p = 0.003)
DPN, n (%)	13 (41.9)	15 (50)	10 (34.5)	0.483
CAN, n (%)	6 (20.7)	11 (39.3)	13 (52)	0.05 (p = 0.017)
DN, n (%)	1 (3.0)	6 (20)	5(17.2)	0.118
DR, n (%)	10 (32.3)	10 (34.5)	7 (23.3)	0.610
ACEI/ARB	7 (21.9)	13 (40.6)	12 (36.4)	0.246
Statin	17 (53.1)	21 (65.6)	22 (66.7)	0.461

Data are shown as mean ± SD, median (interquartile range) or as n (%)

*DM* diabetes mellitus, *WC* waist circumference, *BP* blood pressure, *HbA1*_*C*_ hemoglobin A1_C_, *FPG* fasting plasma glucose, *LDL* low density lipoprotein, *HDL* high density lipoprotein, *hsCRP* high-sensitivity C-reactive protein, *HOMA-IR* homeostasis model assessment-insulin resistance, *ABI* ankle-brachial index, *PWV* pulse wave velocity, *CAN* cardiac autonomic neuropathy, *DPN* diabetic peripheral neuropathy, *DN* diabetic nephropathy, *DR* diabetic retinopathy, *ACEI* angiotensin converting enzyme inhibitor, *ARB* angiotensin receptor blocker

The prevalence of other microvascular complications (DPN, DN, and DR) did not differ among omentin tertiles. In addition, mean serum omentin levels did not differ significantly depending on the presence or absence of microvascular complications (regarding DPN, 496 vs. 559 ng/mL, p = 0.346; regarding DR, 474 vs. 551 ng/mL, p = 0.28; regarding DN, 582 vs 522 ng/mL, p = 0.535) (data not shown).

### Bivariate correlations between omentin and clinical parameters

The results of bivariate correlation analyses between serum omentin, baPWV, and various clinical parameters are shown in Table 3. Serum omentin levels showed significant positive correlations with age (r = 0.3, p = 0.003), baPWV (r = 0.266, p = 0.009), and CAN points (r = 0.327, p = 0.003). HbA1_C_, hsCRP, and HOMA-IR were not correlated with omentin levels. Partial correlation analysis after adjustment for age, mean baPWV, and CAN points revealed that serum omentin levels were positively correlated with CAN points (r = 0.31, p = 0.004) and borderline significantly correlated with baPWV (r = 0.208, p = 0.05).

**Table 3:** Correlation of serum omentin levels and other clinical variables

	Log-transformed omentin			
	Correlation (r)	p value	Partial correlation (r)^b^	p value
Age	0.3	0.003	0.158	0.156
Duration of DM	0.058	0.577		
Body mass index	−0.166	0.105		
WC	0.088	0.455		
Systolic BP	0.051	0.623		
Diastolic BP	−0.036	0.73		
HbA1_C_	0.113	0.271		
FPG	0.045	0.664		
Total cholesterol	0.032	0.76		
LDL-cholesterol	−0.015	0.902		
HDL-cholesterol	0.149	0.148		
Triglycerides^a^	−0.109	0.292		
HsCRP^a^	0.077	0.498		
Creatinine	0.075	0.482		
Insulin (fasting)	−0.131	0.205		
C-peptide (fasting)	−0.114	0.264		
Urinary microalbumin	0.145	0.176		
HOMA-IR	−0.102	0.327		
Mean ABI	−0.029	0.78		
Mean baPWV	0.266	0.009	0.208	0.050
CAN points	0.327	0.003	0.310	0.004

*DM* diabetes mellitus, *WC* waist circumference, *BP* blood pressure, *HbA1*_*C*_ hemoglobin A1_C_, *FPG* fasting plasma glucose, *LDL* low density lipoprotein, *HDL* high density lipoprotein, *hsCRP* high-sensitivity C-reactive protein, *HOMA-IR* homeostasis model assessment-insulin resistance, *ABI* ankle-brachial index, *PWV* pulse wave velocity, *CAN* cardiac autonomic neuropathy

^a^Natural logarithmic transformations were performed before analysis

^b^Partial correlations were computed adjusted for age, mean PWV, and CAN points

### Regression analysis for CAN point

Result of regression analysis for CAN point is shown in Table 4. Univariate regression analysis for CAN point demonstrated that serum omentin and diastolic BP were positively correlated with CAN point and hsCRP was negatively correlated with CAN point. Multiple regression analysis revealed that serum omentin levels, together DBP and baPWV significantly associated with CAN point.

**Table 4:** Linear regression analysis for CAN point

	CAN point			
	Univariate		Multivariate	
	Beta	p value	Beta	p value
Omentin^a^	0.66	0.027	0.61	0.048
Age	0.02	0.09		
Duration of DM	−0.02	0.241		
Body mass index	0.02	0.664		
WC	0.02	0.138		
Systolic BP	0.01	0.068		
Diastolic BP	0.03	0.013	0.04	0.009
HbA1_C_	0.01	0.950		
FPG	0.00	0.415		
Total cholesterol	0.00	0.871		
LDL-cholesterol	0.00	0.586		
HDL-cholesterol	0.00	0.709		
Triglycerides^a^	−0.08	0.701		
HsCRP^a^	−0.30	0.049		
Creatinine	0.03	0.781		
Insulin (fasting)	−0.01	0.693		
C-peptide (fasting)	0.03	0.781		
Urinary microalbumin	0.00	0.867		
HOMA-IR	−0.07	0.336		
mean ABI	−0.18	0.857		
mean PWV	0.00	0.052	0.00	0.042
DPN	−0.18	0.443		
DN	0.27	0.382		
DR	−0.36	0.171		
ACEI/ARB	0.21	0.395		
Statin	−0.09	0.686		

*CAN* cardiac autonomic neuropathy, *DM* diabetes mellitus, *WC* waist circumference, *BP* blood pressure, *HbA1*_*C*_ hemoglobin A1_C_, *FPG* fasting plasma glucose, *LDL* low density lipoprotein, *HDL* high density lipoprotein, *hsCRP* high-sensitivity C-reactive protein, *HOMA-IR* homeostasis model assessment-insulin resistance, *ABI* ankle-brachial index, *PWV* pulse wave velocity, *DPN* diabetic peripheral neuropathy, *DN* diabetic nephropathy, *DR* diabetic retinopathy, *ACEI* angiotensin converting enzyme inhibitor, *ARB* angiotensin receptor blocker

R^2^ = 0.235; F = 5.682; P < 0.001

^a^Natural logarithmic transformations were performed before analysis

## Discussion

In the present study, higher serum omentin levels were associated with a higher prevalence and severity of CAN in patients with T2DM. In addition, elevated serum omentin levels seemed to be associated with arterial stiffness, as assessed by baPWV. On the other hand, there were no associations of serum omentin levels with the presence of other various microangiopathies (DPN, DN, and DR). To the best of our knowledge, this is the first report on the relationships of serum omentin level and all microvascular complications including CAN in patients with T2DM.

Omentin is an adipokine that is mainly expressed in visceral adipose tissue, and has been suggested as a biomarker of metabolic disorders [10]. Several studies have shown that serum omentin levels were negatively correlated with metabolic risk factors and seemed to have anti-inflammatory and insulin-sensitizing effects [8–10]. Moreover, omentin may play a protective role in the cardiovascular system due to its effects on vasodilation, endothelial function and arterial calcification [20, 21]. These actions of omentin may be involved in the pathogenesis of atherosclerosis [22]. Zhong et al. reported that serum omentin levels were lower in patients with coronary artery disease [23]. Analyses of serum omentin levels have been performed mainly in healthy subjects or those with obesity or metabolic syndrome, but rarely in patients with T2DM [24]. Until now, only a few studies have reported associations between omentin and subclinical atherosclerosis (e.g. carotid atherosclerosis) [12, 25]. However, little information is available with respect to the associations of serum omentin levels and diabetic microvascular complications. To the best of our knowledge, no study focusing on the association between omentin and microvascular complications has been published so far. There is now convincing data demonstrating that common pathogenic mechanisms such as inflammation, oxidative stress and altered levels of adipocytokines are related to both microvascular and macrovascular complications [1]. Therefore, we investigated the relationship between omentin and microvascular complications including CAN.

We found that serum omentin levels did not differ between patients with and without each microvascular complication. Also, the prevalence of diabetic microvascular complications (nephropathy, retinopathy and peripheral neuropathy) did not differ significantly among the omentin tertiles. However, the prevalence of CAN showed an increasing trend across the omentin tertiles, and CAN point increased significantly and progressively across the omentin tertiles.

CAN is a significant cause of morbidity and mortality in diabetic patients and is associated with a high risk of cardiac arrhythmia and sudden death, possibly related to silent myocardial ischemia [26, 27]. Few prior studies have reported associations between CAN and levels of adipocytokines (leptin, adiponectin, TNF-alpha) [28]. Although it is not clear why serum omentin levels were positively correlated with the severity of CAN, contrary to the previously established anti-atherosclerotic and cardioprotective roles of omentin, some possible explanations can be suggested. One is that, in response to autonomic dysfunction promoted by endothelial dysfunction, inflammation, oxidative stress, and insulin resistance, the serum omentin level may be increased compensatorily. Generally, recent reports reported reduced omentin levels are related with cardiovascular and metabolic disease, whereas there are some different findings in the literature [13, 29, 30]. Yilmaz et al. reported that omentin levels increased significantly in patients with nonalcoholic fatty liver disease [13]. Another study showed that insulin-sensitizing drugs reduced omentin levels in newly diagnosed patients with diabetes [31]. Also, in a study of asymptomatic prepubertal children, higher serum omentin was associated with a less favorable metabolic profile including features such as insulin resistance and high BP [29]. Kilic et al. reported that plasma omentin levels did not differ between MS patients and control subjects, and in all subjects, omentin was positively correlated with triglyceride levels and negatively correlated with HDL-C levels [30]. They explained that one difference among studies was the technique used to determine omentin levels. Conversely, another speculation is that omentin may pre-date and worsen diabetic CAN, although this is contrary to the known role of omentin as a beneficial adipokine.

In this study, we examined the relationship between omentin and baPWV and ABI as parameters of macrovascular disease. Our study showed that mean baPWV increased significantly and progressively across the omentin tertiles and was significantly positively correlated with omentin level. These results are consistent with those of previous studies. To date, only two studies by Yoo et al. have reported a relationship between serum omentin and arterial stiffening in T2DM. The authors cross-sectionally investigated the association between omentin and arterial stiffness by baPWV in 60 patients with T2DM [24]. Subjects in the highest tertile of serum omentin exhibited the most significant detrimental baPWV values of all the tertiles. Since then, they performed prospective study to investigate the impact of omentin levels on arterial stiffening in 120 patients with T2DM [32]. They observed a much greater increase in serum omentin levels in the group that experienced arterial stiffening. The authors speculated that this paradoxical increase in serum omentin levels in subjects in the increased baPWV group might be a mechanism to compensate for the aggravation of arterial stiffness. In addition, several studies have demonstrated a close relationship between orthostatic hypotension, one of CAN components and arterial stiffness [33, 34]. Therefore, it is possible to speculate that the presence of orthostatic hypotension may contribute to an association of serum omentin with baWV. In our study, four subjects applied to borderline group as a difference of 11–19 mmHg in SBP between lying down and after standing up, whereas one subject applied to orthostatic hypotension as a difference of more than 20 mmHg. In this study, the prevalence of orthostatic hypotension was very low. The contribution of orthostatic hypotension regarding on the association of serum omentin and baPWV may be inconclusive at least in our study. Further prospective studies with larger number of patients having various severity of complications to clarify the role of orthostatic hypotension are needed.

In our present study, significant associations of HOMA-IR or glucose parameters with serum omentin were not detected. Consistent with our study, Hossein-nezhad et al. reported that a significant associations of omentin with HOMA-IR and glucose metabolism were not detected in 81 women [35].

The strengths of the present study are that it is the first report on the association of serum omentin and all microvascular complications, including CAN, in patients with T2DM. Nevertheless, this study has several limitations. This was a cross-sectional study, which therefore could not determine causal relationships between omentin levels, arterial stiffness, and diabetic vascular complications. In addition, there was a potential selection bias because our study population consisted of individuals who had received overall diabetic complication studies; therefore, the characteristics of the present study population may have differed substantially from those of other populations that did not participate in complication studies.

## Conclusions

In conclusion, this present study suggests that serum omentin levels independently associate with CAN in patients with T2DM. In addition, elevated serum omentin levels seem to be associated with arterial stiffness. Future prospective studies with a larger sample size are required to establish a direct relationship between serum omentin levels and the development or severity of CAN.

## Abbreviations

T2DM: type 2 diabetes mellitus
CAN: cardiac autonomic neuropathy
DN: diabetic nephropathy
DR: diabetic retinopathy
DPN: diabetic peripheral neuropathy
baPWV: brachial ankle pulse wave velocity
ABI: ankle brachial index
DBP: diastolic blood pressure
HDL-C: high density lipoprotein cholesterol
CVD: cardiovascular disease
AFT: autonomic function test
HOMA-IR: homeostasis model assessment-insulin resistance
BMI: body mass index
LDL-C: low density lipoprotein cholesterol
hs-CRP: high-sensitivity C-reactive protein
SD: standard deviation
SBP: systolic blood pressure
ACEI: angiotensin converting enzyme inhibitor
ARB: angiotensin receptor blocker
